# Alcoholic Liquors in Tubercular Diseases

**Published:** 1860-03

**Authors:** 


*Alcoholic Liquors in Tubercular Diseases.*

In the October number of the *American Journal of Medical Sciences*, is to be found an able essay by Dr. John Bell, of New York, upon the use of alcoholic liquors in tubercular diseases, or in constitutions predisposed to such disease. The following are the conclusions arrived at:

1. "The opinion so largely prevailing as to the effects of the use of alcoholic liquors, viz., that they have a marked influence in preventing the deposition of tubercle, is destitute of any solid foundation.

2. On the contrary, their use appears rather to predispose to tubercular deposition.

3. Where tubercle already exists, alcohol has no obvious effect in modifying the usual course run by that substance.

4. Neither does it mitigate, in any considerable degree, the morbid effects of tubercle upon the system, in any stage of the disease."

We are glad that the above conclusions coincide with the experiments, and substantiate the views of Dr. Davis, the senior editor, who since 1850, both through the columns of the *North Western Journal*, and in his course of lectures to the numerous classes that have listened to his instruction, has contended that the physiological and pathological effects of alcohol are such as demonstrate that it has no marked influence in preventing the deposition of tubercle, or arresting the course of the diathesis in the least, or that it modifies the effects of the same when it is fully established, to any considerable extent.

